# Supplementary material for: High-Flux Ultrafiltration Membranes Combining Artificial Water Channels and Covalent Organic Frameworks
Source: Membranes (Basel). 2022 Aug 24;12(9):824. doi: 10.3390/membranes12090824 (PMC9503389; doi:10.3390/membranes12090824)
Supplement: Supplementary file 1 [file membranes-12-00824-s001.zip › membranes-1840298-supplementary.pdf]

Supporting Information

High-Flux Ultrafiltration Membranes Combining Artificial Water Channels and Covalent Organic Frameworks

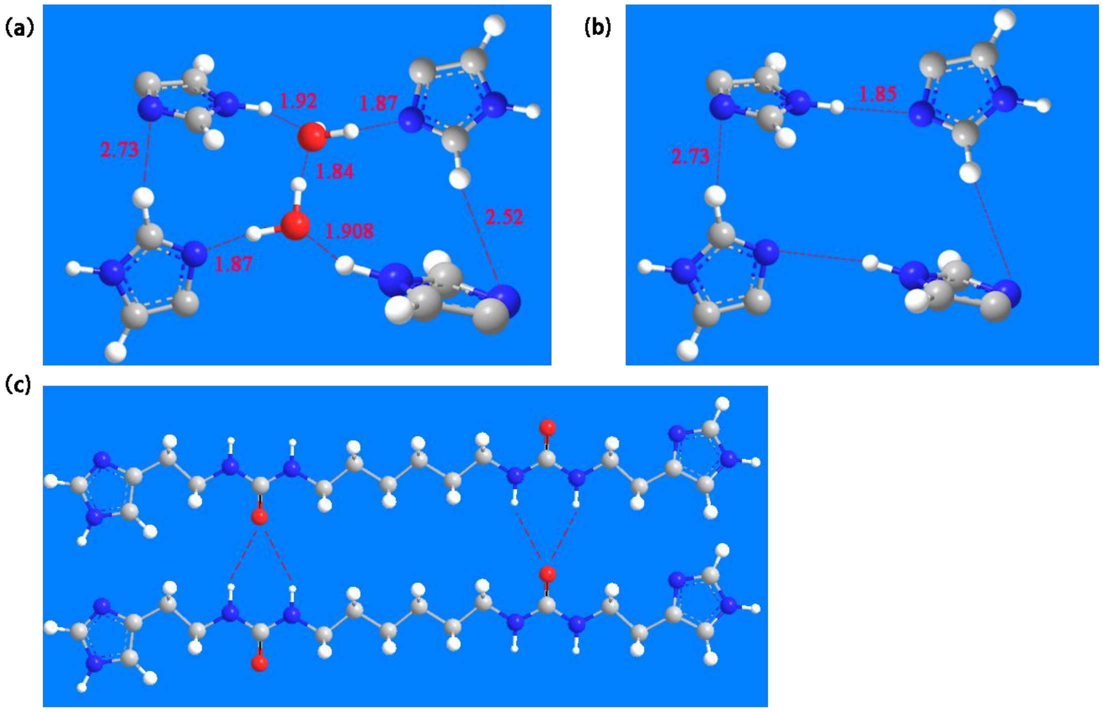

Fig. S1. (a) Water-free I-quartet formation *via* CH···N and NH···N interactions; (b) Water-assisted I-quartet formation *via* CH···N, N···HO, and NH···O-H interactions; (c) HC6H molecules linked via C=O···H (White: H, gray: C, blue: N, red: O, the data is atom distance (Å))

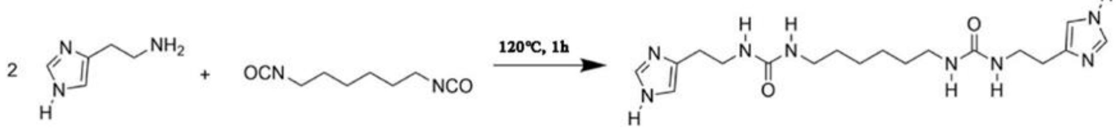

Fig. S2. Synthesis of alkylureido-imidazoles (HC6H) using hexamethylene diisocyanate and histamine

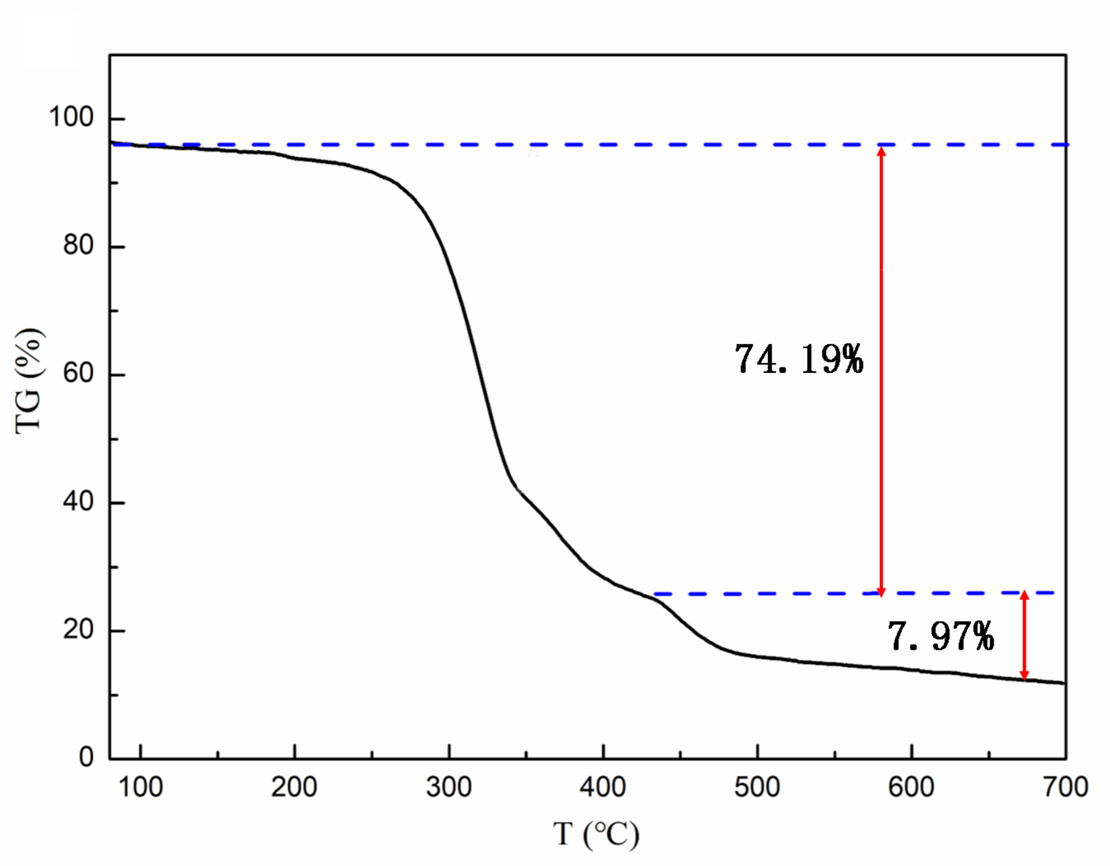

Fig. S3. TGA curves of the synthesized HC6H powders

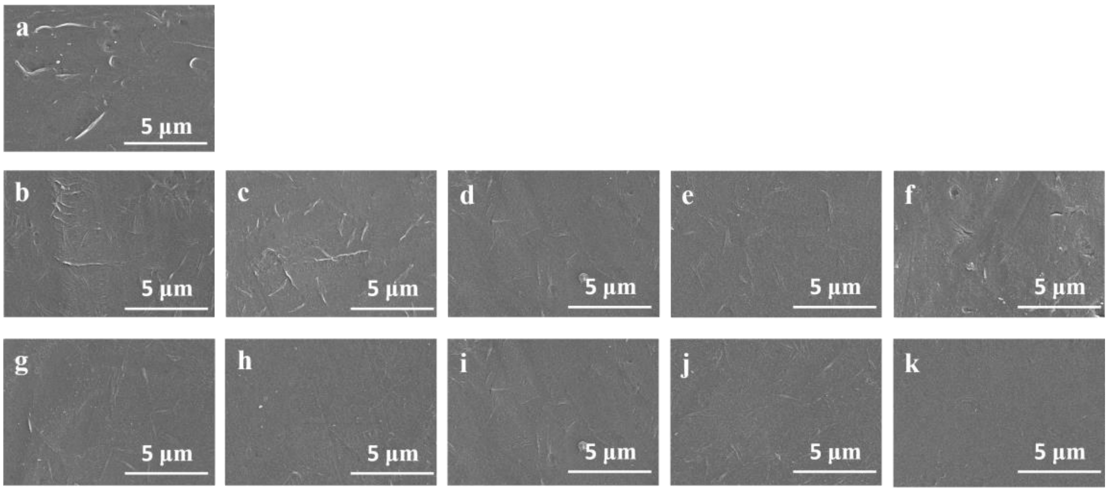

Fig. S4. The SEM images of the upper surfaces of TpPa/HPAN and HC6H-TpPa/HPAN composite matrix membranes prepared at different reaction times and concentrations of HC6H (a: TpPa/HPAN; b: 10 min; c: 15 min; d: 20 min; e: 25 min; f: 30 min; g: 1 mg/mL; h: 1.5 mg/mL; i: 2 mg/mL; j: 2.5 mg/mL; k: 3 mg/mL. The HC6H concentrations for b-f reactions was 2 mg/mL, and the reaction time for g-k reactions was 20 minutes.)

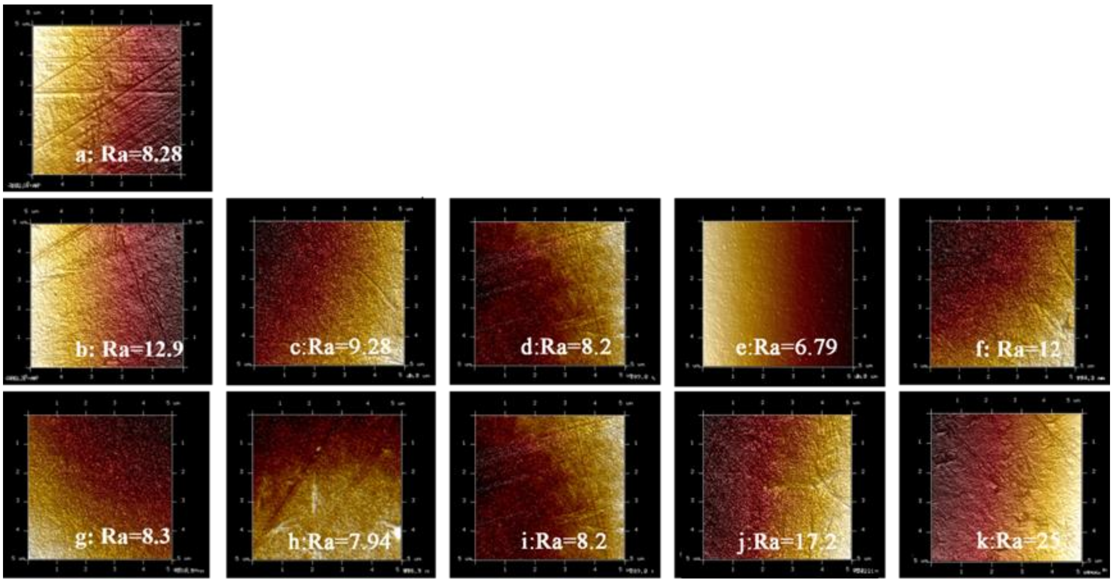

Fig. S5. The AFM images of the upper surfaces of TpPa/HPAN and HC6H-TpPa/HPAN mixed dimensional membranes prepared at different reaction times and concentrations of HC6H (a: TpPa/HPAN; b: 10 min; c: 15 min; d: 20 min; e: 25 min; f: 30 min; g: 1 mg/mL; h: 1.5 mg/mL; i: 2 mg/mL; j: 2.5 mg/mL; k: 3 mg/mL. The HC6H concentrations for b-f reactions was 2 mg/mL, and the reaction time for g-k reactions was 20 minutes.)

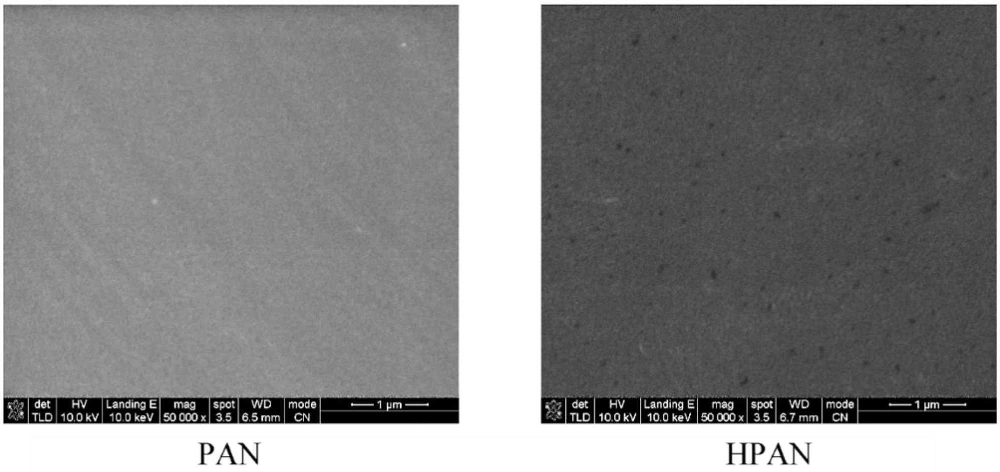

Fig. S6. SEM images of PAN and HPAN membranes.

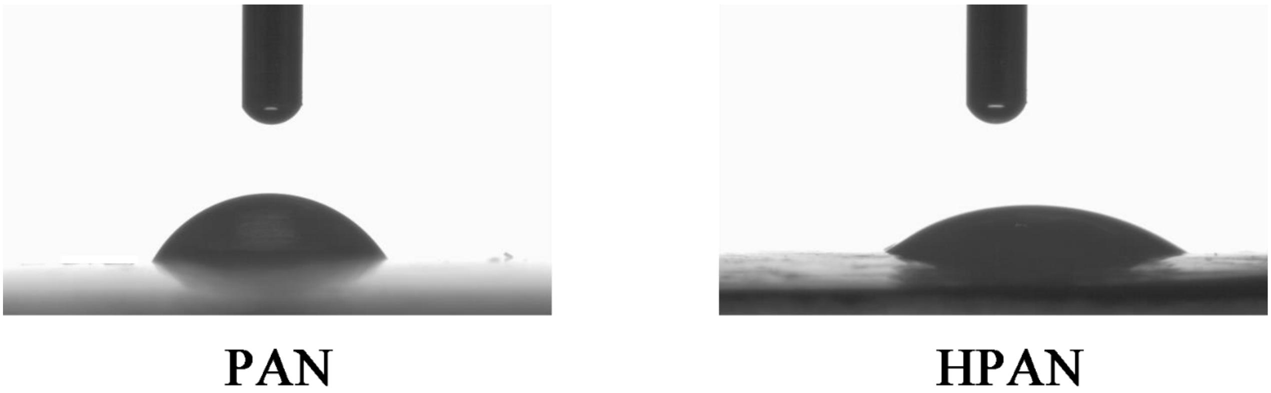

Fig. S7. Water contact angles of the PAN and HPAN membranes

Table S1. The elemental atomic percentage on the membrane surface

	TpPa-HPAN	HC6H-TpPa-HPAN
C 1s	73.2%	72.94%
N 1s	18.33%	20.25%
O 1s	8.48%	6.81%
N/O	2.16	2.97

Table S2. Performance comparison among various membranes towards different dyes rejection

	Dye molecule	Operation pressure(bar)	Flux(L/m2*h*bar)	Rejection (%)	Reference
CMCNa/PP	Congo red	3	9.9	99.8	1
DEA-Modified PA-TFC	Congo red	5	15.74	99.6	2
PAEK-COOH	Congo red	4	25.225	99.8	3
COFs-Go	Congo red	4	30	99.62	4
2D+1D COFs memrbane	Congo red	2	42.8	99.6	5
COFs on polysulfone substrate	Congo red	3	50	99.5	6
COFs-LZU1/PES	Congo red	2	80	99	7
M-TpTD	Congo red	1	120	80	8
PVDF-COFs	Congo red	5	200	99	9
	Rhodamine B			89	
HC6H-TpPa/HPAN composite matrix membranes	Congo red	2	271.76	99.9	This work
	Rhodamine B			96.6	

1. Yu, S.; Chen, Z.; Cheng, Q.; Lü, Z.; Liu, M.; Gao, C., Application of thin-film composite hollow fiber membrane to submerged nanofiltration of anionic dye aqueous solutions. *Separation and Purification Technology* **2012,** *88*, 121-129.
2. Liu, M.; Chen, Q.; Lu, K.; Huang, W.; Lü, Z.; Zhou, C.; Yu, S.; Gao, C., High efficient removal of dyes from aqueous solution through nanofiltration using diethanolamine-modified polyamide thin-film composite membrane. *Separation and Purification Technology* **2017,** *173*, 135-143.
3. Xing, L.; Guo, N.; Zhang, Y.; Zhang, H.; Liu, J., A negatively charged loose nanofiltration membrane by blending with poly (sodium 4-styrene sulfonate) grafted SiO2 via SI-ATRP for dye purification. *Separation and Purification Technology* **2015,** *146*, 50-59.
4. Zhang, X.; Li, H.; Wang, J.; Peng, D.; Liu, J.; Zhang, Y., In-situ grown covalent organic framework nanosheets on graphene for membrane-based dye/salt separation. *Journal of Membrane Science* **2019,** *581*, 321-330.
5. Yang, H.; Yang, L.; Wang, H.; Xu, Z.; Zhao, Y.; Luo, Y.; Nasir, N.; Song, Y.; Wu, H.; Pan, F.; Jiang, Z., Covalent organic framework membranes through a mixed-dimensional assembly for molecular separations. *Nat Commun* **2019,** *10* (1), 2101.
6. Wang, R.; Shi, X.; Xiao, A.; Zhou, W.; Wang, Y., Interfacial polymerization of covalent organic frameworks (COFs) on polymeric substrates for molecular separations. *Journal of Membrane Science* **2018,** *566*, 197-204.

7. Su, Y.-Y.; Yan, X.; Chen, Y.; Guo, X.-J.; Chen, X.-F.; Lang, W.-Z., Facile fabrication of COF-LZU1/PES composite membrane via interfacial polymerization on microfiltration substrate for dye/salt separation. *Journal of Membrane Science* **2021,** *618*.
8. Kandambeth, S.; Biswal, B. P.; Chaudhari, H. D.; Rout, K. C.; Kunjattu, H. S.; Mitra, S.; Karak, S.; Das, A.; Mukherjee, R.; Kharul, U. K.; Banerjee, R., Selective Molecular Sieving in Self-Standing Porous Covalent-Organic-Framework Membranes. *ADVANCED MATERIALS* **2017,** *29* (2).
9. Wu, C.; Wang, X.; Zhu, T.; Li, P.; Xia, S., Covalent organic frameworks embedded membrane via acetic-acid-catalyzed interfacial polymerization for dyes separation: Enhanced permeability and selectivity. *Chemosphere* **2020,** *261*, 127580.
